# Observational measure of implementation progress in community based settings: The Stages of implementation completion (SIC)

**DOI:** 10.1186/1748-5908-6-116

**Published:** 2011-10-06

**Authors:** Patricia Chamberlain, C Hendricks Brown, Lisa Saldana

**Affiliations:** 1 Center for Research to Practice, 12 Shelton McMurphey Blvd., Eugene, OR 97401, USA; 2 University of Miami Miller School of Medicine, 1425 NW 10th Avenue, Miami, Florida 33136, USA

## Abstract

**Background:**

An increasingly large body of research is focused on designing and testing strategies to improve knowledge about how to embed evidence-based programs (EBP) into community settings. Development of strategies for overcoming barriers and increasing the effectiveness and pace of implementation is a high priority. Yet, there are few research tools that measure the implementation process itself. The Stages of Implementation Completion (SIC) is an observation-based measure that is used to track the time to achievement of key implementation milestones in an EBP being implemented in 51 counties in 53 sites (two counties have two sites) in two states in the United States.

**Methods:**

The SIC was developed in the context of a randomized trial comparing the effectiveness of two implementation strategies: community development teams (experimental condition) and individualized implementation (control condition). Fifty-one counties were randomized to experimental or control conditions for implementation of multidimensional treatment foster care (MTFC), an alternative to group/residential care placement for children and adolescents. Progress through eight implementation stages was tracked by noting dates of completion of specific activities in each stage. Activities were tailored to the strategies for implementing the specific EBP.

**Results:**

Preliminary data showed that several counties ceased progress during pre-implementation and that there was a high degree of variability among sites in the duration scores per stage and on the proportion of activities that were completed in each stage. Progress through activities and stages for three example counties is shown.

**Conclusions:**

By assessing the attainment time of each stage and the proportion of activities completed, the SIC measure can be used to track and compare the effectiveness of various implementation strategies. Data from the SIC will provide sites with relevant information on the time and resources needed to implement MTFC during various phases of implementation. With some modifications, the SIC could be appropriate for use in evaluating implementation strategies in head-to-head randomized implementation trials and as a monitoring tool for rolling out other EBPs.

## Background

Moving evidence-based programs (EBP) into routine practice settings is a priority for improving the public's health (National Institutes of Mental Health strategic goal #4) [1,2]. Potential strategies to accomplish this goal have been informed by multi-level conceptual frameworks and heuristic taxonomies that have identified an array of key influences and outcomes that should be considered to achieve successful implementation. For example, Proctor *et al*. [3] identified eight implementation outcomes, including acceptability, adoption, appropriateness, feasibility, fidelity, cost, penetration, and sustainability. Glasgow *et al*. [4] developed a practical, robust implementation and sustainability model (PRISM) that integrates concepts from quality improvement, chronic care, diffusions of innovations, and measures of population-based effectiveness studies of translation. In addition, researchers have developed comprehensive catalogs of the factors shown to affect the success of implementation efforts [4-7]. These comprehensive models and others like them (reviewed in Palinkas *et al*. [8]) tap into an array of social, organizational, and political contexts and influences that are likely to interact with each other and impact implementation outcomes. Such models incorporate common themes that relate to the multi-level nature of implementation, consider that implementation is rolled out in identifiable stages, and identify different processes within implementation stages that may overlap and accelerate or decelerate at different rates [9-11].

In this paper, we describe a tool designed to document progress through implementation stages using a focused observation-based measure of key milestone attainment. The Stages of Implementation Completion (SIC) was developed to measure the progression through implementation stages of an evidence-based program being rolled out in the context of a randomized controlled trial. The measure was not intended to be a checklist or guide for implementing sites, even though the utility of checklists for improving the quality of patient care have been well documented [12]. Rather, the SIC is a measure used to monitor and evaluate the completion of implementation activities, the length of time taken to complete activities, and the proportion of activities completed. The SIC has eight stages with sub-activities within each stage. The eight stages range from initial engagement with the developers to practitioner competency. The SIC is being used to examine the implementation of multidimensional treatment foster care (MTFC), an evidence-based program that is an alternative to residential care for children and adolescents [13].

MTFC has been shown in previous trials to reduce placements in group and institutional settings for youth with severe mental health and behavioral problems [14-16]. It is an intensive multi-component treatment model that requires recruitment and support of community foster homes and provision of an array of mental health and psychosocial support services. As such, implementation is complex and requires a substantial commitment of resources and sustained focus as the agency/site moves through a series of stages to plan for and execute the implementation process. MTFC shares this staged roll out method with other mental health-related evidence-based programs such as multisystemic therapy, a family therapy based model that has been shown to improve outcomes for juvenile offenders. These evidence-based models are highly prescribed in contrast to more organic or gradual methods of implementation that might better characterize less highly specified programs such as wrap-around service models. The development of the SIC was motivated by the need to have a usable and relevant measure of movement through implementation stages that did not add burden to the sites who were already taking on the additional commitments required to implement MTFC. Like other measures of implementation milestones [17], the SIC stages were organized around three overarching implementation phases: pre-implementation, implementation, and sustainability [18].

In this paper, we report on the use of the SIC in the context of an ongoing randomized trial that compares two implementation strategies in county child service systems in California and Ohio. Counties were matched on key characteristics (*e.g.*, population size, percent minority, number of previous placements in residential care), randomized to one of three timeframes (cohorts), and then randomized to one of the two implementation conditions--community development teams (CDT) [19], the experimental condition, or standard individualized implementation (II), the control condition. The II control condition employed the 'as usual' standard consultation package where an MTFC content expert (purveyor) moved the implementation process forward. The standard consultation package included a series of planning/readiness telephone calls, a stakeholder meeting in the individual county/agency, a five-day clinical staff training, weekly case review with video coding and consultation, and periodic site visits. In the CDT condition, in addition to receiving the standard consultation package typically used to implement MTFC, the cohorts of counties participated in peer-to-peer networking during a series of in-person meetings and group telephone calls to share information and strengthen problem-solving skills to overcome barriers of implementation. This was augmented by technical assistance by local consultants versed in state policy and funding streams..

To develop a useful measure for monitoring, evaluating, and comparing both CDT and the II strategies, the SIC was constructed to reflect the same overall stages for both implementation strategies (*e.g.*, there are identical requirements for counties to achieve full credentialing as sustainable programs). Both strategies also contained equivalent activities within the stages, but these activities were sometimes delivered in different ways (*e.g.*, a group peer-to-peer meeting with multiple counties participating in the CDT condition versus a comparably designed meeting delivered to a single county in the II condition). The aim of this paper is to describe the SIC and to present preliminary data on the feasibility and usefulness of the measure as a means to evaluate implementation progress.

## Methods

### Participants and context

Data collection for the SIC is ongoing within the trial to test the relative effectiveness of the CDT and II strategies. All study procedures and informed consent protocols were reviewed and approved by the Center for Research to Practice (CR2P) Institutional Review Board that was awarded a grant from the National Institute of Mental Health to conduct the study. CR2P subcontracts with the California Institute of Mental Health (CIMH), which developed the CDT strategy to implement the CDT condition. Prior to the study, CIMH, acting as a broker, extended an invitation to all California counties to implement MTFC. Based on this invitation, nine counties elected to proceed; these early adopting counties were excluded from the current trial, which focuses on 'non-early adopters' [20]. In addition, eight other 'low need' counties who had fewer than six youth in group care on snapshot days were excluded from the trial because the MTFC model was not thought to be relevant to their service system needs. After three years of operation in California, the study was extended to counties in Ohio. Using procedures in Ohio that were similar to those in California, we excluded one early adopting community and all low need counties. The remaining 38 eligible counties in Ohio were sorted on county size and we then invited 23 counties to participate in random order. Eligible Ohio counties were enrolled using a rolling invitation until 12 counties were recruited. All counties were enrolled that had system leaders who signed a consent form indicating that they were interested in at least considering implementation of MTFC in their county. There are a total of 51 counties from the two states enrolled in the study with 53 study sites participating (two counties had two sites).

In the context of this study, the relative effectiveness of the two implementation strategies being compared includes measurement of the progression through the SIC stages, the duration of progression, and the proportion of activities completed (or skipped) within each of the stages. At this point in the trial, while all counties have been enrolled, several have not had sufficient time to complete the implementation process. Therefore, to illustrate the utility of the SIC, we provide examples of the scoring protocol for three counties who completed (n = 2) or withdrew (n = 1) from implementation. Outcome data comparing the effectiveness of the two strategies will be presented in future reports.

### Development of the SIC measure

During the design phase of the study, the study team, the authors, and J. Reid (CR2P) along with T. Sosna and L. Marsenich from the CIMH, mapped out the stages of implementation based on their experience implementing MTFC in over 70 previous sites. The SIC originally contained 12 stages; however, during the first years of the trial, after applying the SIC to several sites, some activities were eliminated because they were not readily observable or because they were frequently skipped. As more observations of behavior were made, an iterative readjustment process was made with four of the stages being collapsed, eventually resulting in an eight-stage measure; two to seven activities populate each of the stages. Within each stage, observable activities were identified that could be counted as markers or milestones of completion of the stage. In order to minimize bias, an emphasis was placed on including observable activities and on tracking the dates at which those activities occurred; we wanted to structure the measure so that a third-party evaluator who had no investment in a site's progress could reliably score whether an activity had been completed. Second, we wanted to minimize the burden on the site. The SIC measure is completed when the evaluator or researcher codes information such as the date of completion of activities conducted in the normal course of implementing MTFC requiring no input from participants at the setting or site level.

Table 1 shows correspondence of the implementation phase, the SIC stage, activities within stages, and site personnel involvement. As seen there, the SIC is designed to include observation of the participation of agents at multiple levels, from system leaders whose primary involvement typically occurs in the pre-implementation and sustainability phases to practitioners who are typically involved in the implementation and sustainability phases.

**Table 1:** Implementation phases, stages, activities, and participants

Phase	Stage		MTFC Activity	Involvement
Pre-Implementation	1	Engagement	1.1 Date site is informed services/program available* 1.2 Date of interest indicated 1.3 Date agreed to consider implementation	System Leader
	2	Consideration of Feasibility	2.1 Date of first contact for pre implementation planning 2.2 Date first in-person meeting/feasibility call** 2.3 Date Feasibility Questionnaire is completed**	System Leader, Agency
	3	Readiness Planning	3.1 Date of cost/funding plan review ** 3.2 Date of staff sequence, timeline, hire plan review ** 3.3 Date of foster parent recruitment review ** 3.4 Date of referral criteria review ** 3.5 Date of communication plan review ** 3.6 Date of in-person meeting** 3.7 Date written implementation plan complete** 3.8 Date service provider selected	System Leader, Agency
Implementation	4	Staff Hired and Trained	4.1 Date agency checklist completed 4.2 Date first staff hired 4.3 Date Program Supervisor trained 4.4 Date clinical training held 4.5 Date foster parent training held 4.6 Date Site consultant assigned	Agency, Practitioners
	5	Adherence Monitoring Processes in place	5.1 Date data tracking system training held 5.2 Date of first program administrator call	Practitioners, Child/Family
	6	Services and Consultation Begin	6.1 Date of first placement 6.2 Date of first consult call 6.3 Date of first clinical meeting video reviewed 6.4 Date of first foster parent meeting video reviewed	Practitioners, Child/Family
	7	Ongoing Services, Consultation, Fidelity Monitoring and Feedback	7.1 Dates of site visits (3) 7.2 Date of implementation review (3) 7.3 Date of final program assessment	Practitioners, Child/Family
Sustainability	8	Competency	8.1 Date of certification application 8.2 Date certified	System, Agency, Practitioner

Notes: A date of completion is entered for each stage that reflects either (a) the date of completion of the last activity in that stage, keeping in mind that activities may occur in a different order than they are listed, or (b) the date that the site discontinues/quits. The stages and activities could undergo further revisions based on ongoing psychometric analysis. *indicates a variable that is included for duration scoring but not included in the proportion of activities. **indicates activities that are completed as a group for CDT condition and individually for the II condition

## Results

Three scores are derived from the SIC: the number of stages completed; the time spent in each stage (stage duration); and the proportion of activities completed in each stage. The number of stages completed is a simple count of progression through the eight stages; the score is the last stage in which at least one activity was performed. The time spent in each stage was calculated by taking the difference between the date of completion of the first activity in the stage and the date of completion of the last activity in the same stage. Skipped activities are not included in the time calculation. If a site skips the last activity in a stage and completes an activity in a subsequent stage, they automatically moved to the subsequent stage. However, if they later complete the skipped activity, the duration score is adjusted for the original (earlier) stage to include the activity. This allowed durations of the stages to overlap. For sites that completed all eight stages, the final completion date is logged accordingly in stage eight. For sites that chose to discontinue implementation at any point in the process, the discontinue date is logged accordingly in the furthest stage that the site enters. In the case where data are summarized before the stage is complete but a site has not discontinued implementation, the site data are treated as being censored, just as it would in a standard time-to-event or survival analysis [21]. The proportion of activities completed is calculated as the number of activities completed divided by the number of possible activities in each stage. Activities in each stage are ordered based on their logical progression up to the last activity the site completes in the stage or completion of the final activity in the stage. Achievement of either activity indicates completion of that stage.

Although the study is ongoing and therefore final results are not yet available, so far, we have noted several variations in the order that counties move through each stage. For example, we have seen occasions when activities are skipped entirely, and we have observed instances when activities in a later stage precede completion of those in an earlier one (*i.e.*, overlapping). Of the 53 sites enrolled in the trial, all have had sufficient time to complete the pre-implementation phase (stages one to three). Of those, 26 sites remain engaged in the implementation phase (stages four to seven) and three have reached the sustainability phase (stage eight). Three examples of county patterns of completion are shown in Table 2.

**Table 2:** Examples of SIC for three counties

		Site #1		Site #2		Site #3	
**Stage**	**# of Activities**	**Proportion of activities**	**Duration (days)**	**Proportion of activities**	**Duration (days)**	**Proportion of activities**	**Duration (days)**

1	2	100%	8	100%	19	100%	81
2	3	100%	428	100%	118	67%	1
3	8	88%	276	100%	113	38%	83
4	6	83%	30	100%	219		
5	2	50%	1	100%	39		
6	4	100%	29	100%	495		
7	7	86%	328	86%	685		
8	2	100%	111	100%	100		

Table 2 shows that counties one and two completed all eight stages in 1,211 and 1,788 days, respectively. County three discontinued at stage three with a duration score of 165 days. The total proportion scores across stages for counties one and two were 88.4% and 98.3%, respectively, indicating relatively low rates of skipped activities. The large differences in duration by stage are reflective of differences in how the counties approached implementation. For example, county one spent almost two years in the pre-implementation phase, which includes engagement, feasibility assessment, and planning. After that period of contemplation and planning, they moved relatively quickly through implementation stages, taking only 60 additional days before they placed their first youth in MTFC. County one then monitored program fidelity and staff competence and received consultation for just over one year before they applied for and achieved certification, a hallmark of a competent and sustainable program. Certification for MTFC requires meeting a series of nine performance criteria including achieving sustainable enrollment levels and success rates (http://www.mtfc.com). County two moved more quickly through pre-implementation in just over eight months, however, they took nearly four years to achieve competence and sustainability. Finally, county three discontinued implementation efforts during the pre-implementation phase and skipped 7 of the 13 suggested activities in that phase. Wang *et al*. examined the role of county demographic variables and reported county-level predictors of early engagement [22]. A key finding from that study was that system leaders appeared to be most influenced in stage one (engagement) by their objective need for an alternative to group home placements in their county. Counties with positive organizational climates were also more likely to consider implementing MTFC.

## Discussion

Although accelerating the implementation of EBPs into routine practice is a priority, the pace at which this is happening remains frustratingly slow [23]. Little is known about what steps are necessary and sufficient to successfully implement EBPs such as MTFC in the real world. The SIC was designed to track the time it takes to achieve progress milestones, the proportion of those milestones that are completed or skipped, and the completion/lack of completion of eight stages within three phases of implementation.

The SIC shares common elements with a measure of implementation progress that was developed and used by Bergh *et al*. [17] to measure the implementation progress of the kangaroo mother care (KMC) intervention in 65 hospitals in South Africa. As compared to the eight stages in the SIC, the KMC measure includes six stages that describe successive progressions through the implementation process: awareness, adopting the concept, mobilization of resources, evidence of using the practice, routine and integration, and sustainable practice. As in the KMC measure, each of the eight SIC stages relates to a specific implementation milestone. The milestones span the timeframe from the initial engagement stage when the first contact between interested parties occurs through the attainment of program competency.

An advantage of both the KMC and the SIC measures is that no additional effort is required by community participants to generate the data beyond participating in the activities that comprise the usual implementation process. The commitment to implement an EBP typically includes increased demands on resources, such as additional staff training and fidelity monitoring that might stress agency resources. These additional demands often create costs that are not recoverable within available reimbursement streams. Future work with the SIC will focus on specifying these implementation costs.

The current trial compares the effectiveness of the CDT to the II 'usual' implementation strategy that has been used to implement MTFC in more than 70 sites in the United States and Europe since 2002. To date, there has been little research comparing strategies for implementing EBPs in mental health care [11]. The amount of time it takes in each implementation stage has practical and cost implications for implementing sites. The ongoing study will investigate whether there is systematic variation in the counties randomly assigned to the two implementation conditions (CDT or II).

The usefulness of the SIC as an early diagnostic tool is also being examined. In the current trial, we are examining both the effects of skipping activities and the optimal time frames for stage completion relative to two primary outcomes: if and when services to children and families began (*i.e.*, the time to the first MTFC placement), and if and when the program competency is achieved (MTFC certification). Saldana *et al*. [24] found that progression through early stages of implementation during the pre-implementation phase (*i.e.*, time in stage and proportion of activities completed) predicted achievement of the actual provision of services (stage six), suggesting that the SIC could be used as a monitoring guide to provide early feedback to communities about whether they are more or less likely to succeed in implementation.

Several limitations of the SIC measure should be noted. First, the current version of the SIC does not include all relevant information about the implementation process. One planned step in the measurement's development includes the specification of quality indicators. Several of the stages appear to lend themselves to this type of measurement because relevant data are available as part of the usual implementation process such as feedback from participants during staff training and scores from fidelity measures. Ideally, such quality measures would utilize data from multiple perspectives (the community providers and EBP purveyor). Second, the current version of SIC does not measure how widely services are delivered (reach). Such data could be especially important to determine if an EBP is scalable and sustainable over time. A third limitation is that the SIC provides no information on why activities were skipped or on why sites choose to perform activities in a given order. Such information could be useful for improving implementation strategies.

Identifying the next steps in the development of the SIC measure could be relevant to the implementation of other EBPs. Because the SIC has only been applied to MTFC, the universality of the stages has not been evaluated. The specific activities that are indicators of progress in each stage are now relevant only to MTFC. Future research is planned to determine whether these could be developed for other EBPs.

Finally, the psychometrics of the SIC measure are still under investigation. The relationship between the scores generated by the SIC and other validated measures of key features affecting implementation such as organizational climate has not yet been examined, but these analyses are planned within our ongoing trial once data are complete. Further, ongoing evaluation of the reliability and sensitivity of the measure are underway.

## Conclusions

The data generated using the SIC in California and Ohio counties thus far and the potential future utility of the measure for increasing the understanding of the observable stages and activities in the implementation process is promising. It is hoped that the SIC will address a gap in the measurement of implementation progress, and in doing so will help to move the field of implementation science forward.

## Competing interests

PC is a partner in Treatment Foster Care Consultants Inc, a company that provides consultation to systems and agencies wishing to implement MTFC.

## Authors' contributions

The authors contributed equally to this work. All authors have read and approved the final manuscript.
